# Lightening the Load: The Relationship Between Gait and Cognition for Persons Living with Dementia Engaged in a Non-Pharmacological Intervention

**DOI:** 10.3390/brainsci15111214

**Published:** 2025-11-11

**Authors:** Nicholas Tamburri, Cynthia McDowell, Francesca Berthiaume, Carren Dujela, Jodie R. Gawryluk, Denise Cloutier, Mariko Sakamoto, André P. Smith, Debra J. Sheets, Stuart W. S. MacDonald

**Affiliations:** 1 Department of Psychology, University of Victoria, Victoria, BC V8P 5C2, Canada; 2 Institute on Aging and Lifelong Health, University of Victoria, Victoria, BC V8P 5C2, Canada; 3 Department of Geography, University of Victoria, Victoria, BC V8P 5C2, Canada; 4 School of Nursing, University of Victoria, Victoria, BC V8P 5C2, Canada; 5 Department of Sociology, University of Victoria, Victoria, BC V8P 5C2, Canada

**Keywords:** dementia, cognition, gait, music intervention, time-varying covariation

## Abstract

**Objectives**: Relatively little research has explored whether gait and cognition are systematically associated within-persons across time, especially in persons living with dementia (PLwD). Understanding a shared mechanism between gait and cognition may help elucidate effective intervention strategies for promoting cognitive and physiological health in PLwD simultaneously. **Methods**: 33 PLwD enrolled in an 18-month choral intervention employing a measurement-burst design that facilitated up to 9 assessments per person. Three-level multilevel models investigated the time-varying covariation between cognition and gait velocity (indexed using a GAITRite computerized walkway) under both a walk-only and dual-task condition. **Results**: Significant coupling was observed between gait velocity and MMSE (mini-mental state examination) under the dual-task condition, indicating that, on occasions when an individual’s MMSE was one-unit greater than their personal average, there was a corresponding increase in dual-task gait velocity. **Conclusions**: This study highlights a shared within-person mechanism through which improvements in cognition may facilitate physiological advantages.

## 1. Introduction

There is an urgent need to recognize and improve the quality of life and well-being of individuals living with dementia. As dementia progresses, the profound physical, psychosocial, and cognitive impacts are often overwhelming and can result in feelings of isolation, confusion, and distress. Opportunities to engage in activities or develop new relationships are often limited, social connections begin to diminish, and common psychological comorbidities such as loneliness and depression can significantly affect quality of life. Crucially, research has demonstrated that reduced social networks and pronounced feelings of stress and depression are associated with risk of accelerated cognitive decline [1,2] and increased fall risk [3,4] in older adulthood and specifically in individuals living with dementia [5,6,7]. Conversely, increasing evidence also suggests that alleviating such psychological comorbidities in persons living with dementia (PLwD) may facilitate improvements in cognitive function [8,9,10]. While some of these comorbidities may be addressed through pharmacotherapy, there is an ongoing awareness and demand for alternative, non-pharmacological approaches to help mitigate dementia-related symptoms, enhance cognitive and social well-being, and promote physiological function for PLwD.

Non-pharmacological interventions such as art, dance, and music therapy may be effective by providing a sense of belonging, engagement, and connection [11,12,13]. Amongst other benefits, research has demonstrated that various arts-based interventions help to assuage loneliness and negative mood [14,15], enhance quality of life and self-esteem [16], decrease depressive symptoms [11,17], agitation [18] and anxiety [19,20,21], as well as attenuate cognitive decline [9,15]. Non-pharmacological arts-based interventions are therefore being increasingly considered as inexpensive, non-invasive, and effective approaches to help mitigate the negative effects that are often associated with dementia. In fact, the Canadian Dementia Priority Setting Partnership (2017) identified research on non-pharmacological treatments, and whether they can reduce, replace, or be used in conjunction with pharmacological treatments, as a top research priority for Canadians [22].

### 1.1. The Importance of Capturing Variability in Individuals with Dementia

Alongside the growing application of non-pharmacological interventions, there is a commensurate need to evaluate and understand the impact of such approaches using innovative methodology and quantitative rigor. For example, while previous investigations such as randomized controlled trials have demonstrated the benefits of music-based interventions between individuals, there remains a significant gap in understanding the effects of non-pharmacological interventions within individuals. Crucially, dementia is frequently characterized by variable symptom expression; that is, rather than steady, linear changes, PLwD often experience ‘good days and bad days’ in which they are more, or less, symptomatic on some days compared to others [23]. In effect, PLwD experience heightened variability, and assessing within-person fluctuations may be more suitable for monitoring cognitive functioning and symptomatology, relative to emphasizing central tendency paradigms (as are often the focus in randomized controlled trials). It is therefore important to employ research designs that capture this within-person variability and consider individual-level change for PLwD. Variability estimates provide a useful predictive metric for global neural integrity and diminishing brain structure [24] and can enhance our understanding of the dynamic relationship between individual fluctuations in cognitive performance and underlying central nervous system (CNS) health. To this end, additional research is needed to better understand the intricate within-person connections between cognitive health variability and various interconnected processes, shedding light on the factors contributing to both the enhancement and decline of health and well-being in individuals with dementia.

### 1.2. Gait Variability and Cognition, Within-Person Associations

Variability in gait performance (e.g., in velocity, stride time, step length, etc.) has been linked to numerous impairments related to physical health and cognition and may serve as a sensitive indicator of CNS functioning for PLwD [25,26,27]. Previous literature indicates that variability in gait performance is linked to declines in several cognitive domains including executive function, memory, and processing speed [27,28,29], while also being associated with mild cognitive impairment and dementia risk [29,30]. Dual-task gait performance (i.e., where gait is assessed while simultaneously performing a cognitive task) has shown to be especially valuable in identifying individuals undergoing process-graded declines, as it increases the demand on cognitive resources and exposes deficits in systems shared by cognition and gait (e.g., [26,31,32]).

Critically, while the between-person associations among gait and cognition are well explored, both at cross-section and longitudinally (see [33] for a review), little research has investigated how gait variability may be systematically associated, within-persons, with variability in cognition across time. Intraindividual variability (i.e., short-term fluctuations in within-person functioning [34]) and the identification of whether two functional metrics systematically covary across time can only be assessed through longitudinal designs which prioritize multiple repeated measurement-burst occasions per person [35]. One of the strengths of such intensive repeated-measure designs is that it allows researchers to investigate how short-term variation in performance on one measure, relative to a person’s own mean, is related to changes in other repeated variables across time [36]. Investigating the time-varying covariation between gait and cognition is important to identify whether the observed between-person associations of gait and cognition extend to the level of the individual (as within- and between-person effects can often differ in both magnitude and direction; Refs. [37,38]) and elucidate whether within-person positive change in one domain is systematically associated with benefits in the other. For PLwD, identifying a dynamic, within-person link between gait and cognition could help identify a mechanistic pathway by which non-pharmacological interventions targeting the improvement of cognitive and psychosocial health, for example, could confer both cognitive and physiological health benefits.

### 1.3. The Current Study

The current study utilizes longitudinal repeated measures data from PLwD engaged in the Voices in Motion project—an innovative, psychosocial choral intervention that combines music and socialization to enrich memory, reduce isolation and depressive symptoms, and enhance social engagement [9,39]. The Voices in Motion project spanned 18 months across three distinct choral seasons, providing up to nine assessments per participant and allowing for the distinct parameterization of interindividual differences, intraindividual change and variability [34]. Specifically, this study employed three-level multilevel models with person-mean centered predictors to explore the within-person time-varying covariation between cognitive and gait performance, controlling for select individual-differences predictors. Such an approach permits the investigation of within-person change, relative to an individual’s own average performance, and whether within-person global cognitive function fluctuates in tandem with fluctuations in gait velocity across monthly retest sessions. Moreover, we examine this within-person cognitive variability on different gait conditions—specifically, gait under a walk-only and a dual-task—to elucidate how changes in cognitive performance impact gait dynamics in PLwD when under heightened cognitive load. This distinction is pivotal for a comprehensive understanding of the interplay between cognitive and physiological processes, how enhancements in one domain may impact the other, and the importance of cognitive load and resource competition in PLwD. It is expected that within-person increases in cognitive function may favorably affect the coordination and execution of motor tasks, such as walking speed, due to the shared resources involved in both cognitive and motor functions. This study represents a novel addition to the literature, leveraging an innovative methodological paradigm to explore within-person covariation of gait and cognition in PLwD.

## 2. Materials and Methods

### 2.1. Participants

This study was approved by the Human Research Ethics Review Board at the University of Victoria (#20-0329, 8 December 2017). Participants consisted of 33 PLwD (M-age = 79.1; SD = 8.0; range = 57–98 years; 55% female) engaged in the Voices in Motion choral project. Voices in Motion is an intergenerational choral intervention consisting of PLwD and their care partners, as well as local volunteer high school students. Thirty-four PLwD took part in the intervention, however, one individual was excluded from the present analysis due to an inability to complete the majority of assessments. Whereas most participants (88%) were diagnosed with Alzheimer’s disease, a select few individuals with non-AD dementia subtypes (*n* = 4) were also included in the study. The sample was generally well-educated, with most (94%) having obtained a high school diploma or higher, and all were Caucasian.

Voices in Motion participants were recruited through media advertisements (newspaper, radio) and flyers posted at local community organizations (churches, doctors’ offices, community centers). Initial eligibility required PLwD to have physician-diagnosed mild or moderate dementia in addition to a family care partner willing to participate in both the choir and the research assessments. Eligibility was based on self-report and assessed via phone interview. Participants who transitioned to residential care during the intervention were permitted to remain in the study, despite all PLwD initially recruited from community-dwelling contexts. Exclusionary criteria included a prescription of select medications associated with accelerated cognitive worsening (e.g., benzodiazepines; [40,41]) as well as a self-reported physician-diagnosed previous stroke. All participants provided assent at the outset of data collection, which was confirmed on an ongoing basis, and participants were provided several opportunities for questions regarding their participation before consenting.

### 2.2. Procedure

Participants attended up to three choir seasons spanning a total of 18 months. Each choral season was scheduled for approximately 3.5 months, with seasons separated by summer and winter breaks. Choir seasons culminated with a final concert open to the public. The second and third choral seasons comprised new recruits (*n* = 16 and *n* = 4, respectively) as well as returning participants from season one (*n* = 13), with baseline assessments for new participants occurring at the start of the season. During the choir seasons, participants attended weekly choir sessions involving a 1.5 h song rehearsal, followed by a 30 min period of socialization. The weekly choir sessions were held at two local community churches and directed by a professional conductor (BMus, DipEd, MMus). The song repertoire was carefully selected by the conductor to include recognizable, popular songs from the participant’s adolescent and early adult years, songs that elicited strong, positive emotions, as well as songs that promoted hand and other motor movements.

An intensive repeated-measures design was used for the study [42,43] in which various assessments were administered to the participants before the study began, as well as approximately every month (three to four weeks) throughout each choir season. This allowed the researchers to determine whether within-person changes were present in various domains such as in cognitive, physical, and psychosocial functioning, based on participants’ own baseline scores. The assessments included routine social network analysis (SNA), focus groups, and a biopsychosocial (BPS) assessment battery. The latter consisted of physical and neuropsychological assessments, gait mapping, a quality-of-life survey, and cognitive tests that assessed various cognitive domains (including, but not limited to global cognition, executive functioning, reaction time, spatial planning, and short-term memory). Other functional domains measured by the assessment battery included medications, sleep, mood, depression, emotional well-being, socialization, and activities of daily living, among others. Physicians and student research assistants administered the assessment batteries to both PLwD and their care partners separately. Additional information regarding the choral and data collection proceedings can be found in [9].

### 2.3. Measures

#### 2.3.1. Cognitive Functioning

Global Cognitive Function. The Mini-Mental State Examination (MMSE; [44]) a well-validated assessment tool for measuring global cognition, served as the cognitive predictor for the present investigation. The MMSE was verbally administered to participants to determine the degree to which participants were cognitively impaired. Participants were asked questions related to the world (e.g., What is the year?), orientation (e.g., Where are we now?), working memory, and perceptual motor skills. The MMSE is scored out of 30, with a score of 20–24 suggesting mild cognitive impairment, 12–20 indicating moderate impairment, and less than 12 denoting severe cognitive impairment. At intake, the mean MMSE score for the current sample was 20.6 (SD = 5.7, range = 10–29).

#### 2.3.2. Normalized Gait Velocity

Gait velocity, utilized as a proxy for physiological (i.e., CNS) functioning [45,46] was indexed using the GAITRite^®^ (CIR Systems Inc., Sparta, NJ, USA) computerized walkway system [47] with velocity computed by dividing total distance traveled by the ambulation time (indexed in centimeters per second). The GAITRite^®^ system is a computerized walkway embedded with pressure sensors developed to measure temporal, spatial, and gait characteristics including step length and width, cadence, and speed. Participants walked at their normal pace along the walkway in a well-lit environment and while wearing their own comfortable shoes. Practice sessions on the walkway were not permitted prior to testing. Walking commenced 1.5 m prior to the mat and concluded 1.5 m beyond the mat to permit time for acceleration and deceleration. Each sensor pad has an active area of 60 cm square and contains 2304 sensors arranged in a 121.9 × 121.9 cm grid pattern. Sensors are activated under pressure at footfall and deactivated at toe-off, enabling capture of the relative arrangement of footfalls as a function of time. Gait data from the pressure-activated sensors were sampled at 120 Hz and transferred to a computer for subsequent processing using GAITRite^®^ Platinum software (version 4.0) [47]. The GAITRite^®^ system has been reported to be highly valid for measuring temporal and spatial measurements [48,49]. To control for individual differences in height, participant’s original gait velocity (cm/s) was standardized through division by the average leg length (yielding a normalized estimate in units of leg length per second) for each participant.

Gait Conditions. Participants completed two separate walking conditions on the GAITRite^®^ walkway: a walk-only condition, and a dual-task condition (walking performed while simultaneously completing a cognitive task) in order to generate cognitive load. The walk-only task always preceded the dual-task walk. The dual-task condition required participants to count backwards by 7 from 100 while walking on the GAITRite^®^ walkway. Participants completed two complete back-and-forth circuits (four total passes) of the mat, and the four passes were concatenated.

### 2.4. Statistical Procedure

**Figure 1:**
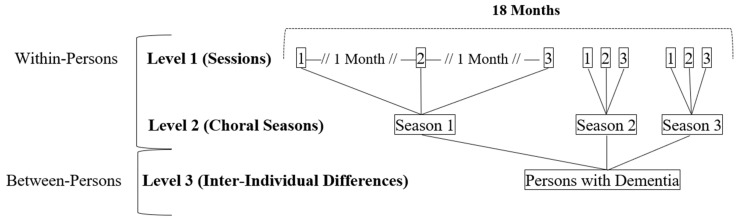
Nested Levels of Change Predicated on the Voices in Motion Longitudinal Intensive Repeated Measures Design.

Multilevel models of change were employed to account for, and separately estimate, nested levels of change inherent within the data structure; three-level models were fit to model within-person change across monthly choral sessions (level 1), choral seasons (level 2) as well as between-person differences (level 3; see Figure 1). Session and seasons were parameterized using precise time-in-study estimates and centered at baseline. Initial unconditional models were fit to derive the intraclass correlation coefficient (ICC), reflecting the proportion of variance explained by the grouping structure and the strong relations of repeated assessments for a given individual (i.e., data dependency; [50]).

Two distinct time-varying covariation models were fit to examine change in cognitive performance (i.e., MMSE), relative to an individual’s own average performance, and whether within-person normalized gait velocity (under the walk-only and dual-task conditions) moved in tandem with cognitive fluctuations across monthly retest sessions (see Equation (1)). Performance on the MMSE was person-mean centered, in which individual’s scores at each time point were subtracted from their own personal mean to derive variability scores and examine the time-varying covariation with gait. By centering in such a manner, the level-1 effect of the time-varying predictor becomes a pure test of the within-person effect, operating locally on the expected outcome, on any single occasion, to quantify fluctuations diverging from the stable person-mean [51]. The β_1i_ slope parameter reflects the rate of linear change in gait velocity across sessions (months in study) and the β_2i_ slope parameter assesses whether increases or decreases in cognitive performance (i.e., MMSE), on a given occasion, are associated with concomitant within-person increases or decreases in gait performance, holding constant linear change over time. The level-3 parameters reflect constant, person-mean effects and include covariates such as average person-mean cognitive performance (γ_001_), chronological age at baseline (γ_002_; centered at 75 years), and sex (γ_003_; coded as male/female). See [9] for further details regarding the structure and benefits of these coupling models (e.g., augmenting statistical power despite a more modest between-subject sample size).

(1) $$\begin{array}{l} Level\;1\;\left(Sessions\right):\;Gait\;{Velocity}_{ijk}=\beta_{0jk}+\beta_{1ijk}{Sessions}_{ijk}+\beta_{2ijk}(MMSE-{\bar{MMSE}}_{ijk})+e_{ijk}\\ Level\;2\;\left(Seasons\right):\;\beta_{0jk}=\delta_{00k}+\delta_{01jk}{Season}_{jk}+U_{0jk}\\ \;\;\;\;\;\;\;\;\;\;\;\;\;\;\;\;\;\;\;\;\;\;\;\;\;\;\;\;\;\;\;\;\;\;\;\;\beta_{1jk}=\delta_{10k}\\ \;\;\;\;\;\;\;\;\;\;\;\;\;\;\;\;\;\;\;\;\;\;\;\;\;\;\;\;\;\;\;\;\;\;\;\;\beta_{2jk}=\delta_{20k}\\ Level\;3\;\left(Persons\right):\;\delta_{00k}=\gamma_{000}+\gamma_{001}{\bar{MMSE}}_{k}+\gamma_{002}{Age}_{k}+\gamma_{003}{Sex}_{k}+V_{00k}\\ \;\;\;\;\;\;\;\;\;\;\;\;\;\;\;\;\;\;\;\;\;\;\;\;\;\;\;\;\;\;\;\;\;\;\;\;\delta_{01k}=\gamma_{010}+\gamma_{011}{\bar{MMSE}}_{k}+\gamma_{012}{Age}_{0k}+\gamma_{013}{Sex}_{0k}\\ \;\;\;\;\;\;\;\;\;\;\;\;\;\;\;\;\;\;\;\;\;\;\;\;\;\;\;\;\;\;\;\;\;\;\;\;\delta_{10k}=\gamma_{100}\\ \;\;\;\;\;\;\;\;\;\;\;\;\;\;\;\;\;\;\;\;\;\;\;\;\;\;\;\;\;\;\;\;\;\;\;\;\delta_{20k}=\gamma_{200} \end{array}$$

All data were modeled in R using the ‘nlme’ package to estimate multilevel models [52,53]. Random effects for each model were examined, and restricted maximum likelihood (REML) was employed as the estimator, as it is known to yield robust parameter estimates with smaller sample sizes [54,55]. Missing data were assumed to be missing at random (MAR). Although MAR is an untestable assumption, we inferentially evaluated whether mean group differences were present between returners and attritors for key modeling variables including age, sex, cognitive status and gait performance. Results suggested that there were no significant group differences among these key variables (all p’s > 0.3); moreover, using all available data, the proportion of missing data across all participants and time points was 13%, 11%, and <1% for gait dual-task velocity, walk-only velocity, and MMSE, respectively.

## 3. Results

### Association Between PLwD Gait and Cognition

Fully unconditioned models revealed that 80% of the total variability in MMSE performance reflected between-person differences, whereas 17% reflected variation within-persons across sessions, and 3% existed within-persons across seasons. Additionally, for gait velocity under the walk-only task, the proportion of variance explained by the grouping structure occurred exclusively between-persons (75%) and within-persons across sessions within seasons (25%), with 0% variation occurring across choral seasons. Likewise, 68% of the variance in the dual-task condition existed between-persons, 32% within-persons across sessions, and 0% across seasons. Such notable within-person variability across sessions suggests that cognitive performance and gait velocity were each fluctuating across month-to-month assessments—providing justification for evaluating the level-1 within-person time-varying covariation between cognition and gait.

Employing three-level multilevel models, and controlling for age and sex, we then assessed whether variability in cognitive performance and gait velocity exhibited significant within-person time-varying covariation by estimating the relationship between within-person fluctuations in cognition and gait across all available months (i.e., sessions nested within seasons). Initially, models were specified to explore random effects of linear change (i.e., whether there were individual differences in rate of change in gait velocity under walk-only or dual-task conditions) and in the coupling parameter (i.e., whether there were individual differences in the association between gait and cognition); however, models failed to converge when estimating these random effects, suggesting modest, non-significant between-person differences in the rate of change or covariation between MMSE and walk-only or dual-task velocity. Accordingly, as specified in Equation (1), final models specified a random effect for intercept only (i.e., allowing individual differences in gait velocity at baseline). Potential moderators, including activities of daily living, affect, depressive symptoms, social support, and time-in-study were initially explored but did not significantly influence the coupling coefficient (all p’s > 0.1). Relatedly, the lack of random effects for the coupling parameter provided initial evidence that, for this sample, individual differences predictors are not likely modulating this effect. Notably, this lack of significant individual differences in the coupling parameter likely stems from several sources, including the modest between-person sample size as well as sample homogeneity (i.e., individuals with a shared progressive neuropathology are known to exhibit functional trajectories that are more homogeneous relative to the general population).

**Figure 2:**
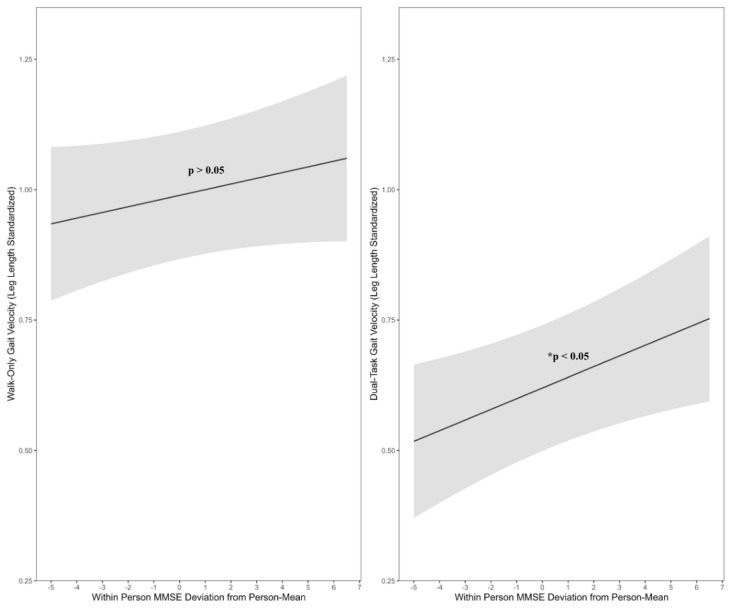
Within-Person Coupling of MMSE on Gait Velocity Under Cognitive Load. Model-derived estimates of predicted change in dual-task gait velocity (standardized as cm/s/leg length) relative to unit changes in within-person deviance in MMSE around their own person-mean. Shaded bands reflect 95% CI’s.

The final three-level models systematically decomposed variation into between- and within-person sources and examined whether within-person variability in cognitive performance was correspondingly related to variation in gait velocity. Consistent with expectations, within-person cognitive performance was significantly associated with gait velocity under the dual-task condition (see Table 1 for a full summary of results for each model). These findings indicate that, irrespective of increases in chronological age and sex (*p* > 0.05), cognitive and gait variability (under the dual-task condition) shared a significant time-varying association across monthly retest sessions. All told, these cognitive and physiological processes were fluctuating together within-individuals (β_2_ = 0.02, 95% CI [0.00, 0.04], *p* = 0.03) such that on occasions when a given individual’s cognitive performance was higher than their personal average, there was a corresponding increase in their normalized gait velocity. Figure 2 highlights this significant main effect, depicting predicted changes in dual-task gait velocity relative to within-person deviance in MMSE around an individual’s own personal mean. In comparison, between-person mean cognitive performance was not significantly associated with gait velocity under either the walk-only or dual-task conditions.

## 4. Discussion

Continued innovation and within-person investigations for PLwD is required to better understand meaningful changes and relationships amongst health domains that may help explain intervention effectiveness and mechanisms underlying the benefits of non-pharmacological approaches for PLwD. The current study examined time-varying covariation (coupled change) by utilizing an innovative longitudinal repeated measures design, providing up to 9 assessments per individual, to evaluate whether cognitive and gait performance moved together within-persons across the course of an 18-month choral intervention. Specifically, we evaluated whether within-person time-varying covariation was present between global cognitive function (indexed using the MMSE) and normalized gait velocity under walk-only and dual-task (i.e., cognitively loaded) conditions.

We demonstrated a significant within-person time-varying association between cognitive performance and gait velocity under the dual-task condition, suggesting that the two health domains were systematically coupled within-persons across sessions (months) in the intervention. During months in which a given individual’s MMSE score was higher, relative to their personal average, there was a corresponding improvement (increase) in their dual-task gait velocity; likewise, a cognitive score lower than their personal average resulted in slower dual-task gait velocity. When proportionalized against the sample average dual-task gait velocity, this amounts to a 2.7% increase (or decrease) in dual-task gait velocity on occasions when MMSE was one unit higher (or lower), respectively. Notably, within-person changes in MMSE resulted in these subsequent changes in gait regardless of stable between-person differences in MMSE, age, or sex. Cognitive performance was not significantly associated, within- or between-persons, with gait velocity under the walk-only task. A lack of significant within-person covariation between walk-only gait velocity and cognition is not entirely unsurprising. As highlighted by our variance decompositions between gait conditions (results from our fully unconditioned models suggested that 25% of variance in walk-only gait velocity was within-persons, relative to 32% in the dual-task condition), walk-only gait velocity seems to be a more stable, dispositional trait that is less sensitive to fluctuations in cognitive functioning. This is supported by previous research which has shown the increased sensitivity of gait performance under the dual-task condition for identifying individuals with cognitive impairment and predicting long-term cognitive decline [26,56,57]. Ultimately, this finding suggests that, of the within-person variance that exists in the walk-only condition, it may be more attributable to stochastic or situational processes (e.g., muscle soreness, inattention, etc.) than it is being driven by fluctuations in cognitive functioning. Indeed, whereas the walk-only gait condition reflects a relatively straightforward daily task, the dual-task paradigm stresses cognition through necessitating the recruitment of additional attentional resources, working memory, problem-solving and decision-making mental processes [26,58]. This multi-component nature of dual-task gait assessments makes them especially valuable at identifying individuals undergoing progressive process-graded declines (e.g., [26,31,32]), and the walk-only condition may simply not be sensitive enough to be influenced by short-term fluctuations in cognition.

The implications of this research are critically important—highlighting the reciprocal relationship of gait and cognition for PLwD. Although walking is considered automatized, when assessing gait under cognitive load, competing cognitive resources can cause perturbations in walking patterns and reduce motor performance. This is particularly true in PLwD, who experience difficulties allocating competing cognitive resources, specifically attention and executive function, between gait and cognitive tasks, often resulting in a slower and more unstable gait [25,26,27,29]. The current findings bolster evidence for this cognitive resource allocation theory, identifying that functional changes in dual-task gait performance fluctuate systematically with global cognitive function within-persons; thus, even slight perturbations in cognition, relative to an individual’s own average, can compromise efficient and effective allocation of attentional and executive processes between the cognitive and motor components of dual-task paradigms. Given that the MMSE represents a reliable, and clinically useful proxy of global cognitive function, the current findings offer a promising insight into how lability in broad cognitive function can serve as a marker of state-vulnerability that can translate to decrements in dual-task gait performance. The clinical implications of this result are predicated on the deleterious consequences of declining dual-task performance. The dual-task paradigm represents an ecologically valid proxy of routine activity; that is, individuals frequently walk while concurrently managing various cognitive demands (e.g., talking, contemplating lists, activities, and schedules). As such, previous research has linked poor dual-task performance to real-world fall risk and frailty [57,59]. Our research highlights the possibility of a shared, underlying mechanism between cognition and gait velocity that manifests when gait is assessed under cognitive load; consequently, an important implication of our research is that interventions capable of enhancing the capacity to manage or reduce cognitive load may yield benefits beyond general cognition, including mitigating the risk of compromised mobility (e.g., fall risk) and improving overall gait functioning.

This aligns with the theoretical framework of the Voices in Motion intervention, which aims to free up cognitive resources by reducing psychological comorbidities (e.g., stress, loneliness, distress), thereby allowing for improved cognitive and functional performance. Indeed, recent results from our lab (see [9]) revealed that participation in the Voices intervention was associated with more a modest cognitive decline for PLwD relative to non-intervention samples; specifically, PLwD declined a modest 1.86 units per annum over their 18-month participation, whereas comparable estimates by meta-analytic work (e.g., [60]) and systematic reviews (e.g., [61]) suggest annualized rates of decline in the MMSE—for PLwD engaged in longitudinal, non-intervention studies—of approximately 3.3 units (90% CI: 2.97, 3.63). Further identified in McDowell et al. [9], on occasions in which individuals reported decreases in negative affect, relative to their personal average, PLwD experienced a corresponding improvement in their cognitive performance. All told, this suggests that engaging in a psychosocial intervention aimed at reducing stress, isolation, and depressive symptoms may attenuate declines by alleviating negative psychological comorbidities. In combination with the current study, these findings highlight a clear lability in within-person signal across cognitive, physiological, and affective domains for PLwD engaged in an arts-based intervention, and that variability across these distinct domains share dynamic within-person associations. This provides a key insight into potential within-person mechanisms, suggesting that the ability to confer positive changes in state-like cognitive, psychosocial, and physiological health in PLwD could facilitate cross-domain advantages. That is, the current study highlights that, given the reciprocal within-person relationship between gait and cognition, interventions that aim to reduce cognitive load or free up cognitive resources may confer advantages for both cognitive and gait control, helping to reduce adverse symptoms and improve quality of life for PLwD.

While this study has several notable strengths, including the use of a measurement burst approach that allows for differentiating stake-like versus trait-like variability and examining longitudinal change [35,46], there are a few limitations. Critically, the Voices in Motion Project did not employ a control group. However, the focus of this study was not to investigate *between-person* differences with a formal control group but rather to examine *within-person* time-varying covariation in which participants effectively served as their own controls. Numerous previous randomized controlled trials have already demonstrated the benefits of non-pharmacological (and more specifically, arts-based) interventions for PLwD; the current investigation sought to examine dynamic within-person change relative to one’s usual level of function rather than stable between-person differences. An additional limitation is the potential influence of order/fatigue effects on tests of gait, as the walk-only condition always immediately preceded the dual-task condition. While this could influence performance, it is also true that this order was held constant throughout the entire 18-month study for every participant, such that any effect of order would be shared across the entire sample. We also did not evaluate the inter-rater reliability of MMSE administration/scoring, which could have erroneously increased variance on the MMSE between- and within-persons across time; however, the MMSE was administered by a small group of intensively trained RA’s—in an attempt to standardize scoring and assessment—and the MMSE has shown good inter-rater reliability in previous studies [62,63]. Finally, having conducted this study in Victoria, British Columbia, our sample lacks sociocultural heterogeneity, potentially affecting the generalizability of our results to populations with different ethnic or socioeconomic backgrounds. Moreover, due to the challenges recruiting PLwD and caregivers to intensive longitudinal research projects, our study operated with a modest sample size, which can pose challenges for model stability—particularly when employing maximum likelihood in multilevel models. Acknowledging this, all models were estimated using REML (known to yield robust parameter estimates with smaller sample sizes) and, despite having a smaller sample size, we nevertheless found significant associations in the hypothesized directions. However, the small sample size did preclude the ability to conduct more in-depth investigations on the relationship between gait and cognition; in particular, we were unable to investigate whether psychosocial factors (e.g., negative affect, stress, etc.) mediated our identified within-person relationship.

The exploration of whether key psychosocial comorbidities mediate the coupling relationship between cognitive and gait performance, using a larger and more heterogeneous sample, is one key avenue for future research that would further inform cognitive resource allocation theory. Moreover, prospective research should also prioritize investigating whether this motor-cognitive relationship is present, and potentially more pronounced, when using more precise measures of cognition, including executive function, working memory, and attention. Due to practical constraints, including high missingness on executive measures, a coupled test of executive functioning and gait could not be evaluated in the current study, despite the relevance of executive functioning in dual-task gait performance. However, these preliminary results showcase domain-general evidence for the relationship between gait and cognition, demonstrating that a reliable, clinically useful proxy of global cognition (i.e., the MMSE) shares systematic within-person associations with dual-task gait. In addition to exploring more sensitive proxies of cognition, future investigations may also investigate whether the identified systematic covariation of dual-task gait and cognition helps to better identify individuals at risk of falls, or whether fall risk is mitigated pursuant to participation in social-cognitive interventions that target a reduction in cognitive load. Given the prognostic utility of dual-task gait performance for assessing functional declines (including fall risk [64]), the current results suggest that even transient stabilization of cognitive functioning could confer benefits to dual-task function; by extension, subsequent longitudinal investigations of non-pharmacological interventions could evaluate whether fall risk is associated with the magnitude of within-person gait-cognition covariation. Such research would also help further contextualize the clinical relevance of the present results. Indeed, given that this is a novel within-person effect which has not been explored previously in the extant literature, ascribing clinical meaningfulness to these significant results remains challenging—all existing clinical norms in gait velocity focus on between-person effects. When contrasted against existing evidence that MMSE can be expected to decline up to 3.63-units per annum in persons living with dementia in non-intervention contexts [60,61], the magnitude of the identified occasion-specific effect suggests that dual-task gait speed could decline up to 10% a year given such patterns of cognitive decline. In future research, continued efforts should explore and further develop within-person norms of physiological outcomes like gait velocity.

## 5. Conclusions

The present study utilized an intensive repeated-measures design, spanning up 18 months and nine assessments per participant, and employed three-level multilevel models with person-mean centered predictors to explore the within-person time-varying covariation between cognitive and gait performance for PLwD engaged in the Voices in Motion choral intervention. Such an approach provides a more nuanced understanding of how cognitive-motor processes are associated within-persons, and how they vary together across time, capturing an important dimension of dementia often overlooked by mean-level analyses. Independent of between-person differences or linear change, we demonstrated a significant within-person time-varying covariation between global cognitive function and gait velocity when individuals are faced with increased cognitive demands. That is, on occasions when an individual’s cognitive functioning was one unit better than their personal average, there was a corresponding increase in gait velocity in dual-task performance. By identifying this within-person coupling association between cognition and dual-task performance, our study highlights the potential importance of cognitive resource allocation as a contributor to functional fluctuations—with worsened general cognition potentially proxying an inability to efficiently or effectively allocate cognitive resources between cognitive and physiological tasks in the dual-task paradigm. This result highlights the importance of identifying programs, such as the Voices in Motion Project, that may transiently stabilize these shared systems through the reduction in deleterious cognitive comorbidities or promotion of affective and motivational engagement, offering a state-level mechanism to support functional performance even for those experiencing progressive cognitive decline. Importantly, due to the ecological validity of dual task performance, identifying strategies to improve cognitive load (e.g., via the reduction in harmful comorbidities) may confer increased protection against adverse physiological events including fall risk and frailty. These findings align with the theoretical framework of arts-based interventions, including Voices in Motion, which aim to enhance cognitive as well as physical well-being in individuals with dementia through promoting social and psychological well-being.

## Figures and Tables

**Table 1:** Within- and Between-Person Effects of Cognitive Performance on Gait Velocity Across Time.

		Walk-Only Task		Dual Task		
	Estimate	Std. Error	95% CI	Estimate	Std. Error	95% CI
Fixed Effects						
Intercept	1.03 ***	0.07	0.89, 1.17	0.73 ***	0.07	0.59, 0.87
TIS (Sessions)	−0.04	0.03	−0.10, 0.01	−0.09 **	0.03	−0.15, −0.03
TIS (Seasons)	−0.08	0.06	−0.20, 0.03	−0.10	0.06	−0.22, 0.02
MMSE (WP)	0.01	0.01	−0.01, 0.03	0.02 *	0.01	0.00, 0.04
MMSE (BP)	0.01	0.01	−0.01, 0.03	−0.00	0.01	−0.02, 0.02
Age	−0.00	0.01	−0.15, 0.01	−0.00	0.01	−0.02, 0.01
Sex	−0.01	0.09	−0.20, 0.18	−0.04	0.10	−0.23, 0.15
TIS × MMSE (BP)	−0.00	0.00	−0.01, 0.01	0.00	0.00	−0.00, 0.01
TIS × Age (BP)	−0.00	0.00	−0.00, 0.00	0.00	0.00	−0.00, 0.00
TIS × Sex (BP)	0.05	0.03	−0.01, 0.11	0.05	0.03	−0.01, 0.12

Note. CI = confidence interval; Time (Sessions) = Time in Study across Sessions (Level-1); Time (Seasons) = Time in Study across Seasons (Level-2); MMSE = Mini Mental State Examination indexing global cognitive function; WP = Within-Person covaration estimate reflecting the time-varying coupling between PLwD cognitive performance and gait velocity occurring month-to-month across sessions within seasons (Level 1); BP = Between-Person individual differences estimate representing the stable person-mean (Level 3).; Sex (Males = 0, Females = 1); Age is baseline age, centered at 75 years. *p < 0.05, ** *p* < 0.01, *** *p* < 0.001.

## Data Availability

Due to the nature of this research, participants in this study did not agree for their data to be shared publicly, so supporting data is not available. For researchers interested in reproduction and extension, please contact the corresponding author for syntax availability and further information. Effort was also put towards developing legacy documents to help individuals build lifestyle/social-cognitive interventions like Voices in Motion within their own communities. Please find these resources publicly available at the Voices in Motion YouTube page, located at: https://www.youtube.com/@voicesinmotionchoirs9841 (accessed on 1 September 2025). These “Train-the-trainer” resources were designed to help facilitate dementia choirs online during the COVID-19 pandemic.

## Abbreviations

The following abbreviations are used in this manuscript:

PLwD	Persons Living with Dementia
MMSE	Mini-Mental State Examination
ICC	Intraclass Correlation Coefficient
CNS	Central Nervous System

## References

[1] Potter G.G., Steffens D.C. (2008). Geriatric depression and cognitive impairment. Psychol. Med..

[2] Wilson R.S., Begeny C.T., Boyle P.A., Schneider J.A., Bennett D.A. (2011). Vulnerability of Stress, Anxiety, and Development of Dementia in Old Age. Am. J. Geriatr. Psychiatry.

[3] Iaboni A., Flint A.J. (2013). The Complex Interplay of Depression and Falls in Older Adults: A Clinical Review. Am. J. Geriatr. Psychiatry.

[4] Stalenhoef P.A., Diederiks J.P., Knottnerus J.A., Kester A.D., Crebolder H.F. (2002). A Risk Model for the Prediction of Recurrent Falls in Community-Dwelling Elderly: A Prospective Cohort Study. J. Clin. Epidemiol..

[5] Fernando E., Fraser M., Hendriksen J., Kim C.H., Muir-Hunter S.W. (2017). Risk Factors Associated with Falls in Older Adults with Dementia: A Systematic Review. Physiother. Can..

[6] Park H.J., Lee N.G., Kang T.W. (2020). Fall-Related Cognition, Motor Function, Functional Ability, and Depression Measures in Older Adults with Dementia. NeuroRehabilitation.

[7] Rapp M.A., Schnaider-Beeri M., Wysocki M., Guerrero-Berroa E., Grossman H.T., Heinz A., Haroutunian V. (2011). Cognitive Decline in Patients with Dementia as a Function of Depression. Am. J. Geriatr. Psychiatry.

[8] Leggieri M., Thaut M.H., Fornazzari L., Schweizer T.A., Barfett J., Munoz D.G., Fischer C.E. (2019). Music Intervention Approaches for Alzheimer’s Disease: A Review of the Literature. Front. Neurosci..

[9] McDowell C., Tamburri N., Smith A.P., Dujela C., Sheets D.J., MacDonald S. (2023). Exploring the Impact of Community-Based Choral Participation on Cognitive Function and Well-Being for Persons with Dementia: Evidence from the Voices in Motion Project. Aging Ment. Health.

[10] Peck K.J., Girard T.A., Russo F.A., Fiocco A.J. (2016). Music and Memory in Alzheimer’s Disease and The Potential Underlying Mechanisms. J. Alzheimer’s Dis..

[11] Cooke M., Moyle W., Shum D., Harrison S., Murfield J. (2010). A Randomized Controlled Trial Exploring the Effect of Music on Quality of Life and Depression in Older People with Dementia. J. Health Psychol..

[12] Hamill M., Smith L., Rohricht F. (2012). Dancing down Memory Lane’: Circle Dancing as a Psychotherapeutic Intervention in Dementia—A Pilot Study. Dementia.

[13] Jeppson T.A., Nudo C.A., Mayer J.F. (2022). Painting for a Purpose: A Visual Arts Program as a Method to Promote Engagement, Communication, Cognition, and Quality of Life for Individuals With Dementia. Am. J. Speech-Lang. Pathol..

[14] Cho H.K. (2018). The Effects of Music Therapy-Singing Group on Quality of Life and Affect of Persons with Dementia: A Randomized Controlled Trial. Front. Med..

[15] Särkämö T., Tervaniemi M., Laitinen S., Numminen A., Kurki M., Johnson J.K., Rantanen P. (2014). Cognitive, Emotional, and Social Benefits of Regular Musical Activities in Early Dementia: Randomized Controlled Study. Gerontol..

[16] Pongan E., Tillmann B., Leveque Y., Trombert B., Getenet J.C., Auguste N., Dauphinot V., El Haouari H., Navez M., Dorey J.M. (2017). Can Musical or Painting Interventions Improve Chronic Pain, Mood, Quality of Life, and Cognition in Patients with Mild Alzheimer’s Disease? Evidence from a Randomized Controlled Trial. J. Alzheimer’s Dis..

[17] Guétin S., Portet F., Picot M.C., Pommié C., Messaoudi M., Djabelkir L., Olsen A.L., Cano M.M., Lecourt E., Touchon J. (2009). Effect of Music Therapy on Anxiety and Depression in Patients with Alzheimer’s Type Dementia: Randomised, Controlled Study. Dement. Geriatr. Cogn. Disord..

[18] Ridder H.M., Stige B., Qvale L.G., Gold C. (2013). Individual Music Therapy for Agitation in Dementia: An Exploratory Randomized Controlled Trial. Aging Ment. Health.

[19] Gómez Gallego M., Gómez García J. (2017). Music Therapy and Alzheimer’s Disease: Cognitive, Psychological, and Behavioural Effects. Musicoterapia En La Enfermedad de Alzheimer: Efectos Cognitivos, Psicológicos y Conductuales. Neurologia.

[20] Irish M., Cunningham C.J., Walsh J.B., Coakley D., Lawlor B.A., Robertson I.H., Coen R.F. (2006). Investigating the Enhancing Effect of Music on Autobiographical Memory in Mild Alzheimer’s Disease. Dement. Geriatr. Cogn. Disord..

[21] Svansdottir H.B., Snaedal J. (2006). Music Therapy in Moderate and Severe Dementia of Alzheimer’s Type: A Case-Control Study. Int. Psychogeriatr..

[22] Bethell J., Pringle D., Chambers L.W., Cohen C., Commisso E., Cowan K., Fehr P., Laupacis A., Szeto P., McGilton K.S. (2018). Patient and Public Involvement in Identifying Dementia Research Priorities. J. Am. Geriatr. Soc..

[23] Rockwood K., Fay S., Hamilton L., Ross E., Moorhouse P. (2014). Good Days and Bad Days in Dementia: A Qualitative Chart Review of Variable Symptom Expression. Int. Psychogeriatr..

[24] Tractenberg R.E., Pietrzak R.H. (2011). Intra-Individual Variability in Alzheimer’s Disease and Cognitive Aging: Definitions, Context, and Effect Sizes. PLoS ONE.

[25] Ijmker T., Lamoth C.J. (2012). Gait and Cognition: The Relationship between Gait Stability and Variability with Executive Function in Persons with and without Dementia. Gait Posture.

[26] Oh C. (2021). Single-Task or Dual-Task? Gait Assessment as a Potential Diagnostic Tool for Alzheimer’s Dementia. J. Alzheimer’s Dis..

[27] Sheridan P.L., Solomont J., Kowall N., Hausdorff J.M. (2003). Influence of Executive Function on Locomotor Function: Divided Attention Increases Gait Variability in Alzheimer’s Disease. J. Am. Geriatr. Soc..

[28] Martin K.L., Blizzard L., Wood A.G., Srikanth V., Thomson R., Sanders L.M., Callisaya M.L. (2013). Cognitive Function, Gait, and Gait Variability in Older People: A Population-Based Study. J. Gerontol. Ser. A Biol. Sci. Med. Sci..

[29] Verghese J., Wang C., Lipton R.B., Holtzer R., Xue X. (2007). Quantitative Gait Dysfunction and Risk of Cognitive Decline and Dementia. J. Neurol. Neurosurg. Psychiatry.

[30] Dodge H.H., Mattek N.C., Austin D., Hayes T.L., Kaye J.A. (2012). In-Home Walking Speeds and Variability Trajectories Associated with Mild Cognitive Impairment. Neurology.

[31] de Oliveira Silva F., Ferreira J.V., Plácido J., Deslandes A.C. (2020). Spatial Navigation and Dual-Task Performance in Patients with Dementia That Present Partial Dependence in Instrumental Activity of Daily Living. IBRO Rep..

[32] Montero-Odasso M.M., Sarquis-Adamson Y., Speechley M., Borrie M.J., Hachinski V.C., Wells J., Riccio P.M., Schapira M., Sejdic E., Camicioli R.M. (2017). Association of Dual-Task Gait with Incident Dementia in Mild Cognitive Impairment: Results From the Gait and Brain Study. JAMA Neurol..

[33] Peel N.M., Alapatt L.J., Jones L.V., Hubbard R.E. (2018). The Association Between Gait Speed and Cognitive Status in Community-Dwelling Older People: A Systematic Review and Meta-Analysis. J. Gerontol. Ser. A.

[34] Nesselroade J., Collins L.M., Horn J.L. (1991). Interindividual Differences in Intraindividual Change. Best Methods for the Analysis of Change.

[35] Sliwinski M.J., Mogle J., Hofer S.M., Alwin D.F. (2008). Time-Based and Process-Based Approaches to Analysis of Longitudinal Data. Handbook of Cognitive Aging: Interdisciplinary Perspectives.

[36] Hoffman L. (2015). Longitudinal Analysis: Modeling Within-Person Fluctuation and Change.

[37] Bielak A.A.M., Cherbuin N., Bunce D., Anstey K.J. (2014). Preserved Differentiation between Physical Activity and Cognitive Performance across Young, Middle, and Older Adulthood over 8 Years. J. Gerontol. Ser. B Psychol. Sci. Soc. Sci..

[38] Kowalski K.A., MacDonald S.W.S., Yeates K.O., Tuokko H.A., Rhodes R.E. (2018). Decomposing the Within-Person and between-Person Sources of Variation in Physical Activity-Cognition Associations for Low-Active Older Adults. Psychol. Health.

[39] Tamburri C., Trites M., Sheets D.J., Smith A.P., MacDonald S.W.S. (2019). The Promise of Intergenerational Choir for Improving Psychosocial and Cognitive Health for Those with Dementia: The Voices in Motion Project. Arbutus Rev..

[40] American Geriatrics Society Beers Criteria Update Expert Panel (2015). American Geriatrics Society 2015 Updated Beers Criteria for Potentially Inappropriate Medication Use in Older Adults. J. Am. Geriatr. Soc..

[41] Rochon P.A., Vozoris N., Gill S.S. (2017). The Harms of Benzodiazepines for Patients with Dementia. Can. Med. Assoc. J..

[42] Rast P., MacDonald S.W., Hofer S.M. (2012). Intensive Measurement Designs for Research on Aging. GeroPsych.

[43] Stawski R.S., MacDonald S.W., Sliwinski M.J. (2015). Measurement Burst Design. The Encyclopedia of Adulthood and Aging.

[44] Folstein M.F., Folstein S.E., McHugh P.R. (1975). “Mini-Mental State”: A Practical Method for Grading the Cognitive State of Patients for the Clinician. J. Psychiatr. Res..

[45] Hausdorff J.M. (2005). Gait Variability: Methods, Modeling and Meaning. J. Neuroeng. Rehabil..

[46] MacDonald S.W.S., Stawski R.S., Diehl M., Hooker K., Sliwinski M. (2015). Intraindividual Variability—An Indicator of Vulnerability or Resilience in Adult Development and Aging?. The Handbook of Intraindividual Variability Across the Life Span.

[47] CIR Systems Inc. (2010). The GAITRite Electronic Walkway.

[48] Bilney B., Morris M., Webster K. (2003). Concurrent Related Validity of the GAITRite Walkway System for Quantification of the Spatial and Temporal Parameters of Gait. Gait Posture.

[49] McDonough A.L., Batavia M., Chen F.C., Kwon S., Ziai J. (2001). The Validity and Reliability of the GAITRite System’s Measurements: A Preliminary Evaluation. Arch. Phys. Med. Rehabil..

[50] Stawski R.S. (2013). Multilevel Analysis: An Introduction to Basic and Advanced Multilevel Modeling. Struct. Equ. Model. A Multidiscip. J..

[51] Hoffman L., Stawski R.S. (2009). Persons as Contexts: Evaluating between-Person and within Person Effects in Longitudinal Analysis. Res. Hum. Dev..

[52] Pinheiro J., Bates D., DebRoy S., Sarkar D., R Core Team (2021). nlme: Linear and Nonlinear Mixed Effects Models, R Package Version 3.1-152. https://CRAN.R-project.org/package=nlme.

[53] R Core Team (2021). R: A Language and Environment for Statistical Computing, Version 4.1.1.

[54] McNeish D. (2017). Small Sample Methods for Multilevel Modeling: A Colloquial Elucidation of REML and the Kenward-Roger Correction. Multivar. Behav. Res..

[55] Singer J.D., Willett J.B. (2003). Applied Longitudinal Data Analysis: Modeling Change and Event Occurrence.

[56] MacDonald S.W.S., Hundza S., Love J.A., DeCarlo C.A., Halliday D.W.R., Brewster P.W.H., Lukyn T.V., Camicioli R., Dixon R.A. (2017). Concurrent Indicators of Gait Velocity and Variability Are Associated with 25-Year Cognitive Change: A Retrospective Longitudinal Investigation. Front. Aging Neurosci..

[57] Muir S.W., Speechley M., Wells J., Borrie M., Gopaul K., Montero-Odasso M. (2012). Gait Assessment in Mild Cognitive Impairment and Alzheimer’s Disease: The Effect of Dual-Task Challenges across the Cognitive Spectrum. Gait Posture.

[58] Sheridan P.L., Hausdorff J.M. (2007). The Role of Higher-Level Cognitive Function in Gait: Executive Dysfunction Contributes to Fall Risk in Alzheimer’s Disease. Dement. Geriatr. Cogn. Disord..

[59] Yeo J., Tay L., Pua Y.H., Mah S.M., Tay E.L., Wang M.X., Ng Y.S. (2024). Single and Dual Task Gait Speed in Frailty Screening of Community-Dwelling Older Adults. J. Prim. Care Community Health.

[60] Han L., Cole M., Bellavance F., McCusker J., Primeau F. (2000). Tracking Cognitive Decline in Alzheimer’s Disease Using the Mini-Mental State Examination: A Meta-Analysis. Int. Psychogeriatr..

[61] Breitve M.H., Chwiszczuk L.J., Hynninen M.J., Rongve A., Brønnick K., Janvin C., Aarsland D. (2014). A Systematic Review of Cognitive Decline in Dementia with Lewy Bodies versus Alzheimer’s Disease. Alzheimer’s Res. Ther..

[62] El-Hayeck R., Baddoura R., Wehbé A., Bassil N., Koussa S., Abou Khaled K., Richa S., Khoury R., Alameddine A., Sellal F. (2019). An Arabic version of the mini-mental state examination for the Lebanese population: Reliability, validity, and normative data. J. Alzheimer’s Dis..

[63] Feeney J., Savva G.M., O’Regan C., King-Kallimanis B., Cronin H., Kenny R.A. (2016). Measurement error, reliability, and minimum detectable change in the Mini-Mental State Examination, Montreal Cognitive Assessment, and Color Trails Test among community living middle-aged and older adults. J. Alzheimer’s Dis..

[64] Muir-Hunter S.W., Wittwer J.E. (2016). Dual-task testing to predict falls in community-dwelling older adults: A systematic review. Physiotherapy.

